# Compassion Fatigue Among Critical Care Nurses and Physicians: A Scoping Review

**DOI:** 10.1002/nop2.70410

**Published:** 2025-12-23

**Authors:** Mohamad Al Nakhal, Angela Massouh, Nuhad Y. Dumit, Charles R. Figley

**Affiliations:** ^1^ Hariri School of Nursing American University of Beirut Lebanon; ^2^ Tulane University, School of Social Work New Orleans Louisiana USA

**Keywords:** burnout, compassion fatigue, critical care healthcare providers, critical care nurse, secondary traumatic stress

## Abstract

**Introduction:**

The continuous engagement with critically ill patients leaves healthcare providers vulnerable to Secondary Traumatic Stress (STS), consequently developing Compassion Fatigue (CF). Nurses' compassion is crucial for healing and is associated with improved care delivery. Hence the purpose of this review is to evaluate the literature related to CF among Nurses and Physicians in the Critical Care area, with an emphasis on the prevalence of burnout (BO), STS and CF.

**Design:**

A scoping review.

**Methods:**

Two main electronic databases, namely Ovid Medline and CINAHL, were used to identify the relevant studies published. Google Scholar, PubMed and hand searching were used to scan the grey literature and otherwise missed peer reviewed literature. Following the title and abstract review, full‐text articles were screened for relevance, and study data were extracted. A narrative approach to synthesising the literature was used.

**Results:**

There is a lack of consensus on conceptualisation and operationalisation of CF. The predictors of CF vary among studies. Most of the studies reported a low to moderate level of CF. Only 2 studies reported high scores of BO, STS and CF.

**Conclusion:**

This paper has highlighted numerous gaps which necessitate further investigations. Despite increased recognition of CF in the healthcare field, its impact and preventive interventions remain limited.

**Clinical Relevance:**

Addressing CF among critical care providers is vital for improving the well‐being of providers, ensuring better patient care, and encouraging healthy and safe work environments.

## 1 Introduction

Nurses' work‐related stress is higher in certain specialties than in others due to the patients' increased psychological and physiological needs for compassionate care (Jin et al. 2021). This stress, particularly evident in critical care settings, can result in indirect trauma, as a result of repeated exposure to trauma survivors, ultimately giving rise to psychological conditions such as Secondary Traumatic Stress (STS), Compassion Fatigue (CF), or Vicarious Trauma (Figley 1995; Tarshis and Baird 2019). Critical care nurses are more vulnerable to traumas, which can be psychologically, physiologically, and emotionally draining (Milligan and Almomani 2020). Nurses and physicians frequently encounter critical patients' conditions, which can lead to STS, a psychological condition stemming from experiencing someone else's suffering, when left untreated, may evolve into CF. This vulnerability arises from caring for chronically and terminally ill patients, making ethical decisions, managing with medical errors, facing communication challenges, and addressing the needs of patients' relatives (Curtis et al. 2014; Epp 2012; Todaro‐Franceschi 2013). Critical care nurses and physicians experience moderate to high levels of STS and are particularly susceptible to CF (Perregrini 2019; Salimi et al. 2020). In the United States, CF affects 40% of registered nurses (Perregrini 2019) and 60% of emergency medicine physicians report experiencing STS (Shanafelt and Dyrbye 2012).

Compassion Fatigue refers to a combination of emotional, physical, and psychological exhaustion resulting from prolonged exposure to compassion stress, caregiving, and empathy (Figley 1995; Sorenson et al. 2017; van Mol et al. 2015). It comprises two components: Burnout (BO) and STS (Balch et al. 2009; Stamm 2002)‐ yet CF remains a distinct phenomenon. BO is characterised by emotional exhaustion, depersonalisation, negative attitudes, reduced personal satisfaction, and sadness, Whereas STS is associated with fear and work‐related trauma (Peters 2018). In contrast Compassion Satisfaction (CS) refers to the fulfilment that caregivers derive from helping others and feeling valued in their professional role (Stamm 2002). Compassion Satisfaction is associated with positive emotions gained from effective trauma care and serves as a protective factor against CF (Al Barmawi et al. 2019; Ruiz‐Fernández et al. 2020; Stamm 2012).

Compassion Fatigue is an emerging concept in the Arab world, and it has not yet been thoroughly examined in Lebanon. In a systematic review of studies from the Arab region reported intermediate to high levels of CF among healthcare personnel (Elbarazi et al. 2017). For instance, Jordanian critical care unit nurses, particularly those working in emergency departments, had low to average level of CS and CF (Al Barmawi et al. 2019). Regarding physicians, although BO has been extensively studied, limited research has explored CF among this provider group (Baer et al. 2017; Garcia et al. 2014; Shanafelt and Dyrbye 2012; Shenoi et al. 2018).

A major consequence of CF, BO, and reduced CS is depersonalisation which leads to diminished empathy, heightened stress, depressive symptoms, physical exhaustion and ultimately impaired nurse–patient interaction. The outcomes contribute to increased medical errors, reduced life‐saving success, and lower quality of care (Balch et al. 2009; Balinbin et al. 2020; Cypress 2011; Maslach and Jackson 1981; Rudolph et al. 1997; Shanafelt et al. 2010; Trzeciak et al. 2019; Winters 2018).

Addressing CF among critical care providers is therefore vital to enhance their mental, emotional and psychological well‐being, promoting excellent patient care, and foster healthy and safe work environments. Doing so can provide valuable insight to cultivate healthier, more collaborative critical care teams and ultimately ensuring sustainable, high‐quality care for critically ill patients.

## 2 Methodology

**FIGURE 1:**
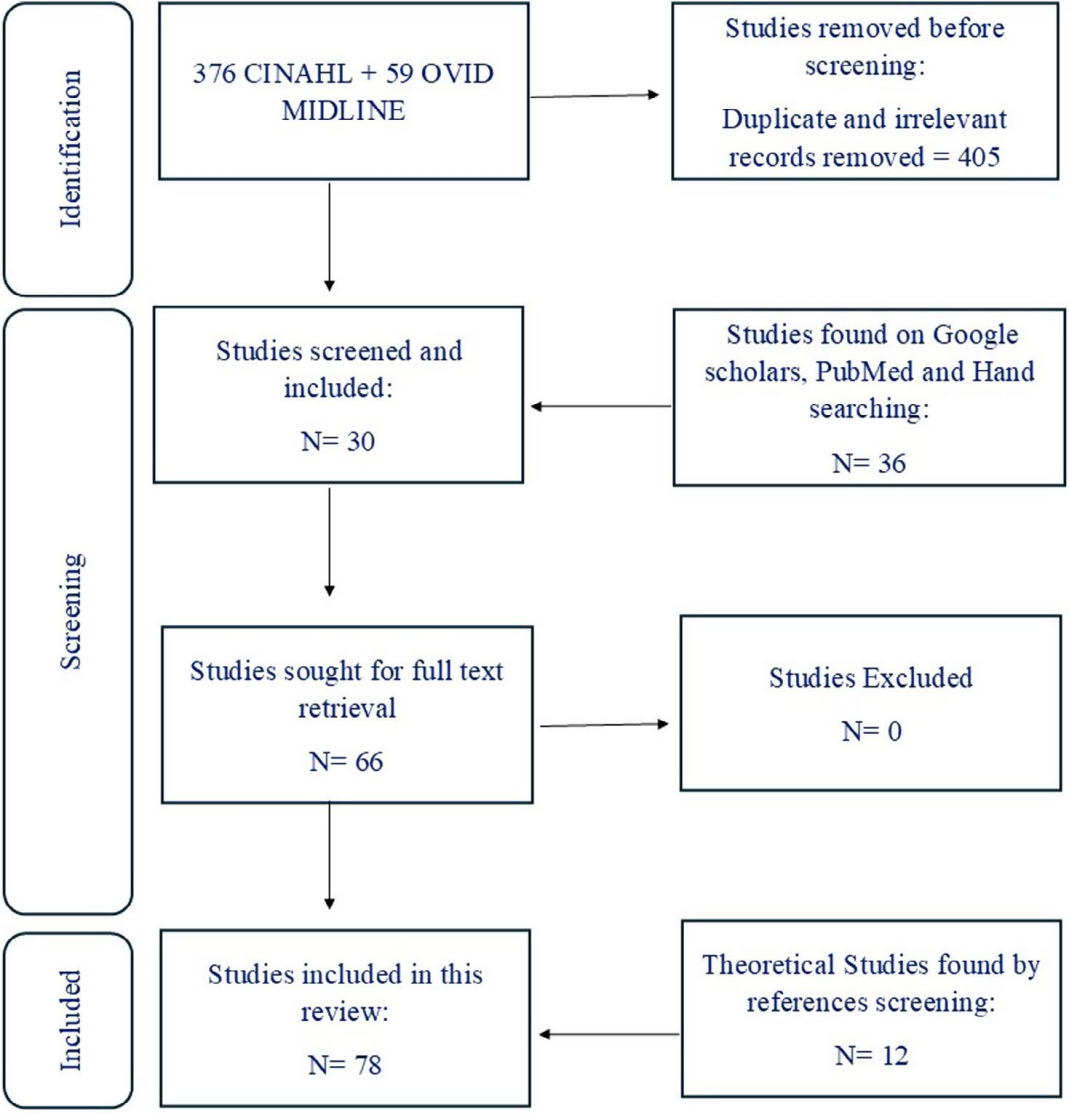
Flow diagram of search strategy.

A scoping review enables the exploration of broad, emerging, or complex research topics (Tricco et al. 2018). Therefore, it is considered an appropriate design to address the aim of this review. The purpose of this scoping review is to map the existing literature on CF and to highlight knowledge gaps that can guide future study, regardless of the study designs included. Structurally, this scoping review follows the five‐stage framework set out by Arksey and O'Malley (2005) which provides a systematic and rigorous approach to examining literature (Davis et al. 2009) by mapping current evidence, summarising research findings, and identifying that warrant further exploration. The five stages are: (1) identifying a research question, (2) identifying relevant literature, (3) selecting literature, (4) charting data, and (5) reporting findings. The search strategy is depicted in the flow diagram below (Figure 1).

### 2.1 Stage 1: Identifying a Research Question

The adopted question for this scoping review is: what does literature detail about CF and CS among critical care nurses and physicians?

Although used interchangeably and indiscriminately, CF is an overarching phenomenon that encompasses both BO, an occupational stressor that arises from workplace strain, and STS which is an acquired trauma that evolves from feeling sufferer's suffering. The aim of this study was to review both theoretical and empirical literature about what is known about CF and CS among critical care nurses and physicians and identify gaps that need further investigation to advance this area of research.

### 2.2 Stage 2. Identifying Relevant Publications

Two main journal databases were used to conduct this review, Ovid Midline and CINAHL. Additional searches were also conducted on PubMed and Google Scholar to ensure broader coverage of the literature. The search was conducted and included articles published from 2008 to 2023. The search was limited to English‐language publications.

The search strategy combined MeSH terms where applicable and keywords. Search terms were organised into three main concepts and combined using Boolean operators “OR” and “AND” as follows:

*Concept 1: Compassion Fatigue, Burnout and Secondary Traumatic Stress*

- MeSH terms: Compassion Fatigue/; Burnout, Professional/; Stress Disorders, Post‐Traumatic/
  ○ Keywords: secondary trauma*; secondary traumatic stress; secondary traumatization*; vicarious trauma*; vicarious traumatization; burnout; career burnout; occupational burnout; professional burnout; moral injur*; PTSD; post traumatic stress disorder; stress disorder post traumatic; stress disorders post‐traumatic; empathy fatigue; trauma exposure response

*Concept 2: Healthcare Setting*

- MeSH terms: Critical Care Nursing/; Critical Care/; Intensive Care Units/
  ○ Keywords: critical care nursing; intensive care nursing; critical care; intensive care; intensive care surgical; surgical intensive care

*Concept 3: Healthcare Professionals*

- MeSH term: Physician/
  ○ Keywords: physician*; doctor*; medical staff; medical practitioner*; resident*; intensivist*; attending; nurse*; nursing staff

The three concepts were then combined using the “AND” operator: (Concept 1) AND (Concept 2) AND (Concept 3). For instance: (compassion fatigue OR secondary trauma* OR burnout OR vicarious trauma* OR moral injur* OR PTSD) AND (critical care OR intensive care OR ICU) AND (physician* OR doctor* OR nurse* OR intensivist*).

Further papers were included by ancestry method through reference list screening of selected articles. Studies were included if they (a) examined CF or CS among critical care nurses and/or physicians, (b) were published in English, and (c) used an empirical (peer reviewed publication) or theoretical approaches (review or theoretical studies). Articles were excluded if irrelevant to the CF among nurses and physician concept, not in English, not in tertiary medical facility, a conference abstract.

### 2.3 Stage 3. Publication Selection

CINAHL yielded 376 results, whereas Midline Ovid produced 59 results. Initially duplicates were identified and removed using EndNote (version 20). The first author independently screened titles and abstracts for potential relevance based on inclusion/exclusion criteria Potentially relevant articles were retrieved for full texts screening. Screening was conducted independently by two reviewers; discrepancies were resolved through discussion and consensus to ensure rigour and reduce selection bias. A total of 30 empirical articles were found relevant on these 2 databases. Additionally, 36 other articles were found by Google Scholar alert, PubMed and hand searching of reference lists. Twelve theoretical articles were included as well in this paper found by hand searching and references screening.

Finally, a total of 66 empirical articles and 12 theoretical articles met the inclusion criteria and were included in this review. The study selection process was summarised in a PRISMA‐ScR flow diagram. All articles reviewed in this scoping review are presented in Tables [Link], [Link], [Link], [Link].

### 2.4 Stage 4. Charting Data

Articles were reviewed and key points data from the included studies were charted in the table. Extracted data included, Author, Year, Country, Sample, Measurement tool, Design, Main findings. The data extraction was conducted by the first researcher and reviewed by the research team. To validate the data extraction process, 8 random publications of the included studies, almost 10%, were cross‐checked by a second reviewer from the research team to ensure accuracy and consistency. References were cited by EndNote (version 20). Following the PRISMA‐ScR standards (Tricco et al. 2018), no formal assessment of quality was conducted in this study, as the intention was not to exclude any papers based on quality appraisal.

### 2.5 Stage 5. Data Synthesis

Due to the variety of designs and heterogeneity of the studies found, a narrative synthesis of findings was adopted in this review. Studies found were tabulated and summarised then a content analysis of the data was performed based on the table data. Findings were categorised into themes and then compared with other studies from another settings. This review followed the PRISMA‐ScR evidence‐based guidelines to ensure methodological transparency and reproducibility and help authors improve quality and completeness of reporting in this scoping review.

## 3 Results

A total of 70 articles were analysed in this study varying between theoretical and empirical. In the review of the literature, 43 cross sectional studies, descriptive and correlational, were found in addition to 4 mixed methods studies. Participants in these studies were in majority critical care and emergency room nurses and physicians. Thirty‐five out of 47, almost three‐quarters 74.5%, reported scores of CF about their studies' participants. Half of these studies, 17 studies, reported a low level of CF and BO among three participants. Eight out of these 17 studies were conducted in US (Abou Hashish and Ghanem Atalla 2023; Aslan et al. 2022; Calegari et al. 2022; Faiza Hameed et al. 2022; Hegney et al. 2014; Jakimowicz et al. 2018; Juniarta et al. 2023; Kelly and Lefton 2017; Lopez et al. 2022; Meadors and Lamson 2008; Mooney et al. 2017; Storm and Chen 2021; Weintraub et al. 2016, 2020; Wu et al. 2016; Young et al. 2011).

The other half, 16 studies, were conducted in developing countries with robust presence of Arabic and middle east countries, showed a moderate level of CF and low to moderate level of BO (Ageel and Shbeer 2022; Al Barmawi et al. 2019; Alharbi et al. 2019, 2020a, 2020b; Cicek Korkmaz and Gokoglan 2023; Delle Donne et al. 2023; Ma et al. 2022; Ndlovu et al. 2022; Panagou et al. 2023; Pehlivan Saribudak et al. 2023; Racic et al. 2019; Ruba Sam et al. 2023; Salimi et al. 2020; Subih et al. 2023; Unlugedik and Akbas 2023; Zeng et al. 2023). Only 2 studies report high scores of BO and STS and thus CF, conducted among Critical care nurses in USA and South Korea (Jeong and Shin 2023; Monroe et al. 2020).

The majority of the studies in this review reported moderate to high CS (Ageel and Shbeer 2022; Al Barmawi et al. 2019; Alharbi et al. 2019, 2020a, 2020b; Calegari et al. 2022; Delle Donne et al. 2023; Gribben et al. 2019; Hegney et al. 2014; Jakimowicz et al. 2018; Juniarta et al. 2023; Kelly and Lefton 2017; Lopez et al. 2022; Meadors et al. 2009; Monroe et al. 2020; Mooney et al. 2017; Nasser Ali et al. 2022; Ndlovu et al. 2022; Panagou et al. 2023; Racic et al. 2019; Richardson and Greenle 2020; Ruba Sam et al. 2023; Salimi et al. 2020; Storm and Chen 2021; Weintraub et al. 2016, 2020; Wu et al. 2016; Young et al. 2011).

Only 4 studies reported a low level of CS and these were conducted in China, Pakistan, Egypt and Jordan (Abou Hashish and Ghanem Atalla 2023; Faiza Hameed et al. 2022; Ma et al. 2022; Subih et al. 2023; Zeng et al. 2023).

This review revealed six themes, namely: CF Conceptualisation, CF operationalisation, CF Predictors and consequences, CF Management and Nurses' perspective about CF.

### 3.1 Theme 1: CF Conceptualisation

There is a lack of consensus in theoretical literature regarding the definition and nomenclature of CF. Terms such as CF, STS, BO, and Vicarious Trauma are often used interchangeably or without clear distinction, leading to confusion. While this overlap reflects the interrelated nature of these conditions, it also obscures their unique characteristics.

Joinson (1992) was the first to discuss CF and defined it as a distinct kind of BO that is related to care‐giving professions, notably nursing. Figley (1995) unlike Joinson, distinguished between BO and CF and defined CF as a kind of post‐traumatic stress disorder (PTSD) related to the mental and emotional expenses of caring for others and practical substitute to “STS”.

According to Valent (2002), CF may exist on a continuum prior to or following BO. Thomas and Wilson (2004) identified three different traumatic outcomes: CF, STS, and vicarious traumatization and characterise CF as a distinct outcome. CF was defined as a gradual and cumulative process caused by extended, intensive interaction with patients that surpasses a nurse's endurance limit (Coetzee and Klopper 2010). Whereas Jenkins and Warren (2012) characterise it as the unavoidable result of dealing with a traumatised (the critically ill patient), and the other of whom is negatively impacted by the first's traumatic event (the nurse in critical care), occurring unexpectedly as opposed to the gradual onset of BO due to ongoing exposure to stressors. On her turn, Stamm provided a model describing helping others as having two sides, a good one called “CS” and a negative one identified as CF, which consists of two components, BO and STS: CS, BO, and STS. Stamm (2002, 2005, 2010).

### 3.2 Theme 2: CF Operationalisation

The lack of consensus in defining CF has resulted in the adoption of multiple questionnaires for its assessment. The Professional Quality of Life (ProQOL‐V) questionnaire was used in most of the reviewed studies, with a lesser number using ProQOL‐IV or the Compassion Fatigue Short Scale (CFS) and Compassion Fatigue and Satisfaction Self‐Test (CFST). The various versions of ProQOL reflect the still‐evolving knowledge of CF, with ProQOL‐V including dimensions for CF (BO and STS) and CS. ProQOL‐IV, assesses three domains: CS, BO, and CF/STS. On the other hand, CFS includes BO and Vicarious trauma as component of CF, and CFST is the original long instrument from which ProQOL was developed.

### 3.3 Theme 3: CF Predictors and Consequences

Literature identifies various predictors of CF, generally categorised into demographic, personal, and organisational factors. However, the findings are often contradictory. For instance, while several studies suggest that being female is a predictor of CF, others report no significant correlation between gender and CF or compassion satisfaction (CS) scores. Similar inconsistencies are observed with variables such as age and years of experience. Similar inconsistencies are observed with variables such as age and years of experience. Some studies have established correlations between CF and patient outcomes, yet others have failed to replicate these results. Moreover, certain variables such as anxiety and depression have shown both positive and negative correlations with CF across different studies. A few investigations have also proposed novel predictors—such as nurse–physician collegiality—though these findings have yet to be confirmed through replication.

A strong correlation existed between CF and Post Traumatic Stress Disorder suggesting that those two concepts overlap (Meadors et al. 2009). Similarly, a moderate to strong positive correlation exists between CF and stress (feeling of distress, work related stress, peritraumatic psychological stress, Moral distress), STS (Amin et al. 2015; Kelly and Lefton 2017; Nasser Ali et al. 2022; Uzun et al. 2022; Weintraub et al. 2016, 2020) (Maiden et al. 2011) and witnessing the death of a child (Amin et al. 2015; Meadors et al. 2009).

Moreover, being single, BO scores, personal health issues, colleagues, not talking about stress, emotional depletion, in‐hospital teaching, participating in administrative activities (e.g., documentation) were found to be determinants of CF. In contrast, age (older generation nurses), being married, having a child, having long years of experience, not feeling depressed or emotionally depleted, being “on call”, staying over shift, being recognised, high job enjoyment and satisfaction, exercising, socialising, ethnicity (Hispanics and Indian) were all significant predictors of CS (Amin et al. 2015; Aslan et al. 2022; Delle Donne et al. 2023; Kelly and Lefton 2017; Meadors et al. 2009; Nasser Ali et al. 2022; Ruba Sam et al. 2023; Weintraub et al. 2016, 2020).

Anxiety, tension, lack of compassion, disconnection, bad coping skills, and general negative attitudes are common symptoms of CF among healthcare providers (Milligan and Almomani 2020). CF was found as a mediator between resilience and both anxiety and depression (Jo et al. 2020). Strong positive correlations were found between nursing sensitive indicators (CAUTI, CLABSI and pressure injuries) and CF (Anglade 2014; Maiden et al. 2011). Two mixed method studies found that nurses who intended to leave or resign have higher CF scores in comparison to their colleagues who chose to stay (Christianson et al. 2023; Maiden et al. 2011).

### 3.4 Theme 4: CF Management

Coping strategies positively predict CS and negatively predict CF (Abou Hashish and Ghanem Atalla 2023; Al Barmawi et al. 2019; Calegari et al. 2022; Weintraub et al. 2020). As well, Level of resilience significantly predicted the rating of CS, BO, and STS. Regression model indicated that resilience as a predictor explained 66% of the CS, 26% of the BO and 15.4% of the STS variance (Alharbi et al. 2019). On the other hand, two studies showed a significant relationship between Spiritual wellbeing and CF, with spiritual well‐being significantly predict CF among nurses and critical care nurses specifically (Ariapooran and Abdolmaleki 2023; Unlugedik and Akbas 2023). Moreover, Cumulative Stress Debriefing attendance demonstrates a correlation and reduction in symptoms related to CF (Arbios et al. 2022). Furthermore, two RCTs were done to test the effect of emotional regulation training sessions and short‐ and long‐term Compassion Fatigue Resilience Program CFRP on nurses' ProQOL score (Kharatzadeh et al. 2020; Pehlivan Saribudak et al. 2023). Both, program and sessions, positively affected CS, however, show no effect on CF.

### 3.5 Theme 5: Nurses' Perspective on CF

Nurses recognised that CF could be handled with self‐care although they were unsure how to do so in their everyday working lives. Permission from themselves and others, namely nursing culture; organisation; managers, appeared to be the key for enabling nurses to continue providing the compassionate care to patients. Nurses described CF as “bruises in the soul” that fade away but leave scares that may affect patients later on if not treated (Andrews et al. 2020; Gustafsson and Hemberg 2022; Jakimowicz et al. 2018). Furthermore, a mixed method study highlighted that critical care nurses don't deliver compassionate care but competent care (Milligan and Almomani 2020).

## 4 Discussion

This review provides a comprehensive review of the available literature on CF among healthcare providers working in critical care settings. Of the 47 empirical studies, almost 80% of the studies (*n* = 38), were conducted in the past 5 years, post COVID, reflecting the growing attention of to CF in critical care. This interest is paralleled by evidence of serious consequences, including increased rates of emotional exhaustion, reduced empathy, and compromised patient care outcomes (Alharbi et al. 2020b; Peters 2018). Unaddressed CF contribute to higher turnover intentions, absenteeism, and long‐term psychological distress threatening the healthcare systems stability and the patient care quality (Zhang et al. 2022).

A substantial proportion of studies were conducted in the Arab countries and the MENA region, providing a more globally representative perspective than Western‐centric research. Furthermore, studies spanned countries with varying income levels, offering insights across diverse demographic and professional groups. However, all studies were in English language, which poses a risk of excluding relevant non‐English research.

CF manifest in critical care nurses as difficulty focusing, poor judgement, disturbing imagery, desperation, tiredness, anger, and irritability (Devilly et al. 2009; Negash and Sahin 2011), yet, no consensus exists on a single definition for CF. Some studies considered CF as distinct concept, others overlapping with STS and BO. This definitional inconsistency limits research mostly to cross‐sectional descriptive and correlational designs. Only a few experiments including randomised controlled trials, quasi‐experimental studies, and longitudinal studies have examined interventions (Kharatzadeh et al. 2020; Arbios et al. 2022; Pehlivan Saribudak et al. 2023; Asadollah et al. 2023; Egami and Farrar Highfield 2023).

Methodological limitations were widespread, lack of interventional research methods, lack of standardised questionnaires to assess for CF, using a non‐validated questionnaire, self‐reported questionnaire, low response rate, cluster randomisation, potentially led to biased findings and exaggerated responses and low statistical power. Furthermore, despite extensive research on BO among physicians, CF among doctors remains under explored.

Regional and cultural factors shape CF experiences in the MENA region. Levantine countries (Jordan, Lebanon) and Egypt differ from Gulf countries in cultural norms, education systems, nurse–patient interactions, and resources, influencing CF. Local nurses often embody community‐centered care, emphasising family engagement (Elbarazi et al. 2017). Conversely, foreign nurses in the face dual pressures of acculturation and maintaining cultural identity which complicates acculturation and contributes to emotional strain (El‐Jardali et al. 2011).

Also, educational and systemic disparities exacerbate CF. Nursing programs in the Levant and Egypt while culturally contextualised, lack resources and international linkages present in Gulf programs (Aiken et al. 2014). Gulf systems prioritise evidence‐based practice with significant funding (Alshammari et al. 2019) creating a mismatch of demands and capabilities across regions.

Nurse–patient ratios emphasise CF burden. The American Association of Critical‐Care Nurses (AACN 2024) recommends a 1:2 ratio in geriatric critical care wards to assure excellent treatment while reducing BO. However, Levantine ratios are higher. In Jordan, ICUs ratios fluctuate between 1:4 at university medical centers to 1:5–1:8 in Governmental institutions (Shuriquie et al. 2008), while Lebanon face critical care nurses shortages (Alameddine et al. 2020). These circumstances increase workloads, burnout, and CF prevalence.

Furthermore, support networks for nurses vary across regions. Levantine hospitals adequate resources, resulting in BO and high attrition (Alharbi et al. 2020b) while Gulf nations are investing in nurse psychological wellness initiatives, social support, and professional growth (Alosaimi et al. 2013). Cultural stigmas in the Levant and Egypt, where CF is seen as weakness further hinder obtaining the necessary care (Hamdan Mansour et al. 2020).

Finally, professional status differences affect CF. In Egypt and the Levant, nurses may have limited recognition, hiring and retention opportunities as well. Expat nurses in Gulf, are often valued, yet this might reinforce hierarchies and local marginalisation among native nurses (Almutairi et al. 2015).

Although no formal quality appraisal was performed, consistent with scoping review methodology, a critical reflection on the included studies revealed notable variability in methodological rigour. Overall, definitional inconsistencies, methodological limitations, and regional disparities hinder the development of evidence‐based interventions. Methodological challenges additionally restrict the generalisability of the findings. Limitations are summarised in the table below (Table 1). Future research should use participatory action research (PAR), engaging bedside staff to co‐develop interventions reflecting lived experiences. In addition, healthcare organisations should implement standardised staffing, anti‐stigma initiative, and equitable resources distribution. A multifaceted, frontline‐centered approach in policy, practice, and research is critical to protect healthcare workers' mental health, building resilience, and ensuring high‐quality patient care worldwide.

**TABLE 1:** Analytical integration across reviewed studies.

Theme	Key findings	Contradictions/variations	Research gaps/limitations
Definition of CF	Fatigue Irritability Poor judgement Distressing imagery	Some studies treat CF as distinct Others as STS/BO	Lack of consensus Hinders intervention development
Study designs	Mostly cross‐sectional studies Descriptive studies Correlational studies	Few experimental/longitudinal studies	Need RCTs Quasi‐experimental Longitudinal designs
Measurement tools	Varied Instrument Self‐reported questionnaires	Non‐validated, inconsistent	Bias, social desirability Low statistical power Comparison hindrance
Population and region	MENA and Western studies	CF experiences differ by culture Nurse status Resources	Few studies on physicians Non‐English publications excluded
Impact on staff and patients	Emotional exhaustion Reduced empathy Turnover Absenteeism	Severity varies regionally	Need longitudinal studies on interventions' effectiveness
Organisational and cultural factors	Workload Nurse–Patient Ratios Education CF Cultural Stigma Hierarchical structures	Gulf: ○ High resources, hierarchical Levant/Egypt: ○ Low resources ○ Familial care model	Limited research on support systems Anti‐stigma programs
Interventions	Some experimental studies show positive effects (Kharatzadeh et al. 2020; Arbios et al. 2022)	Small samples Inconsistent methods	Lack of scalable Culturally adapted interventions

## 5 Conclusion

This paper has identified several theoretical and empirical gaps in the study of CF, that warrant further investigation. Despite increased recognition of CF within the medical field, understanding its underlying effects, impacts on related factors, and effective interventions to mitigate its impact remains limited.

Theoretical gaps are evident in the absence of consensus regarding the conceptualisation of CF. The interchangeable and indiscriminate use of CF alongside concepts such as STS, Vicarious Traumatization, and BO reflects conceptual ambiguity and the interrelated nature of these phenomena.

Empirically, although extensive, a notable gap persists in interventional and preventive studies aimed at addressing CF. Moreover, the employment of multiple assessment tools with varying dimensions complicates the comparison of findings across studies. While numerous predictors of CF have been explored, results are frequently contradictory and lack replication. Methodological limitations have also been noted in the reviewed studies, particularly in the few that employ interventional designs. Finally, while numerous studies have focused on CF among nurses, studies among physicians are relatively limited.

In conclusion, addressing these theoretical and empirical gaps is crucial for advancing our understanding of this phenomenon and developing effective strategies for its prevention and management. Future research should aim to clarify conceptual boundaries, standardise measurement tools, and design robust interventional studies to enhance healthcare professionals' well‐being and prevent declines in the quality of patient care.

## Funding

The authors have nothing to report.

## Clinical Resources

PRISMA for Scoping Reviews: http://www.prisma‐statement.org/Extensions/ScopingReviews; Professional Quality of Life version V ProQOL‐V manual: https://proqol.org/proqol‐manual.

## Conflicts of Interest

The authors declare no conflicts of interest.

## Supporting information


**Table S1:** Summary table of included cross‐sectional studies.

**Table S2:** Summary table of included mixed method studies.

**Table S3:**Summary table of included qualitative studies.

**Table S4:** Summary table of included experimental studies.

## Data Availability

The data that supports the findings of this study are available in the Supporting Information of this article.
