# Supplementary material for: Compassion Fatigue Among Critical Care Nurses and Physicians: A Scoping Review
Source: Nurs Open. 2025 Dec 23;12(12):e70410. doi: 10.1002/nop2.70410 (PMC12723723; doi:10.1002/nop2.70410)
Supplement: Supplementary file 1 — Table S1: Summary table of included cross‐sectional studies. [file NOP2-12-e70410-s001.docx]

**Table 1. Summary table of included Cross-Sectional studies**

| **Author** | **Country** | **Sample** | **Measurement tool** | **Overall findings** |
| --- | --- | --- | --- | --- |
| (Meadors & Lamson, 2008) | USA | 167 HCW in PICU, NICU, PEDS | - STS Scale STSS - Pro-QOL-V - Impact of Events Scale-Revised | - Intrusion, avoidance, and arousal scales strongly correlate with CF. BO is linked to CF and overlaps with PTSD. High CS is associated with lower STS and BO. CS, BO, PTSD, and STS account for 70% of CF, with STS being the largest factor. Physicians (62.5%) and child-life specialists (61.5%) report moderate to high CF after recent patient loss. CF and BO symptoms decrease over time following traumatic events. |
| (Young et al., 2011) | USA | 70 nurses between heart vascular ICU and IMC (intermediate care) | - Pro-QOL-V | - 60% of IMC nurses had a high level of CS, with 84% experiencing low BO and only 24% reporting average STS. In contrast, 82% of ICU nurses had an average CS level, 64% showed average BO, and 44% had average STS. Nurses in the HV-IMC reported significantly higher levels of CS. |
| (Hegney et al., 2014) | Australia | 132 critical care nurses in tertiary hospital | - ProQOL-5 - DASS-21 | - CS is linked to lower anxiety and depression, while CF correlates with higher levels. About 20% of nurses show depression risk due to elevated CF symptoms. Recognizing these risk profiles can help managers identify treatment needs. However, no relationship was found between ProQOL, DASS, and the intention to leave. |
| (Amin et al., 2015) | India | 129 NICU nurses | - Pro-QOL-V - Perceived Stress Scale (PSS14) | - Most nurses experience moderate to high stress. PSS14 is negatively correlated with CS and positively correlated with BO and STS. Age and relationships with doctors explain over 5% of the variance in perceived stress. |
| (Carole Branch, 2015) | USA | 296 pediatric health care providers (nurses, physio, physicians, inhalations) | - Pro-QOL-V | - Cardiology unit healthcare staff have higher CS than those in other units. PICU staff report a significantly higher BO compared to staff in any other unit, with PICU nurses showing lower CS scores and higher BO and STS scores. |
| (Sacco et al., 2015) | USA | 221 critical care RNs and LPNs | - Pro-QOL-V | - Compared with male nurses, female nurses reported significantly higher CS scores. Nurses aged 40 to 49 had significantly lower CS and higher BO than older nurses. Nurses aged 20 to 29 reported significantly higher STS levels than their older colleagues. Single-acuity unit nurses had significantly higher CS than those in mixed-acuity units, which had significantly higher BO and STS. Nurses experiencing a recent management change reported significantly lower mean CS scores. CS was positively and significantly correlated with level of education and age. Nurses under a change in nursing management reported significantly higher levels of BO. |
| (Weintraub et al., 2016). | USA | 433 faculty neontologist | - CF and Satisfaction Self-Test for Helpers (CFST) | - CF, BO, and CS prevalence rates were 15.7%, 20.8%, and 21.9%, respectively. A strong positive correlation between CF and BO had a shared variance of 58%. Negative correlations between BO and CS, as well as CF and CS, had shared variances of 54% and 38%. CF prevalence was 15.1%, with females at higher risk. Significant CF determinants included female gender, emotional depletion, clinical distress, co-workers, personal health issues, and neglecting self-care. High CS predictors included being Hispanic, having additional family members, over 10 years of experience, and recent "on call" clinical activity. |
| (Wu et al., 2016) | Canada/US | Oncology nurses 549 | - Pro-QOL-V - Abendroth Demographic Questionnaire | - There were no statistical differences in CS, BO, and STS between the US and Canada. Younger nurses experienced more STS. A cohesive team environment helped buffer negative feelings |
| (Kelly & Lefton, 2017) | USA | 726 ICU nurse from hospital with DAISY recognition program &  410 nurses from hospitals without | - Pro-QOL-V - A job-related question | - Nurses in the study reported their jobs as satisfying, enjoyable, and stressful, with 40% considering leaving the profession. They experienced low BO, moderate STS, and moderate CS. Stress showed a significant positive correlation with BO and STS, while satisfaction and enjoyment had significant negative correlations with both. Sex, age, staying over shifts, enjoyment, and satisfaction positively correlated with CS. The “DAISY” recognition program negatively predicted BO and positively predicted STS and CS. |
| (Mooney et al., 2017) | USA | 102 nurses from oncology and ICU | - Pro-QOL-V | - The majority of nurses have high levels of CS and low to average levels of BO and STS. ICU nurses and females reported significantly lower CS and higher BO levels. Male nurses reported lower CF than females. Years of experience negatively impacted CF, while age showed a negative correlation with both CF and BO. The number of children correlated negatively with BO. |
| (Gribben et al., 2019) | USA | 475 Pediatric critical care fellows and attending physicians | - CF and Satisfaction Self-Test for Helpers (CFST) | - A strong positive correlation exists between CF and BO scores. There are strong negative correlations between CS and BO scores, as well as between CF and CS scores. Female sex is positively correlated with CF. The largest contributing factor to the CF score is the BO score |
| (Jakimowicz et al., 2018) | Australia | 98 critical care nurses | - Pro-QOL-V | - Mean scores for CS, BO, and STS were within the average range (23-41). The majority reported high CS, average BO, and low STS, with 33.3% having average CF. Years of experience and tenure positively correlated with CS, while age, years of experience, and tenure negatively correlated with BO. No associations were found between STS and other factors. Nurses with 5 to 15 years of experience had the highest BO levels. BO and STS negatively correlated with CS, while STS and BO positively correlated with CF. Nurses with postgraduate degrees had significantly higher CS. Place of work significantly predicted STS and CS, while years of tenure significantly predicted BO. |
| (Sano et al., 2018) | USA | 174 registered nurses in the level III and IV NICUs | - Pro-QOL-V - Multi Questionnaires | - Multiple instruments used alongside PROQOL included the Strength of the Nurse-Infant/Family Relationship, nurse-physician relations subscale, Self-Compassion Scale, STS Scale, and Oldenburg BO Inventory. Self-compassion mediated the relationship between the nurse-infant/family relationship and CF, STS, and BO when nurse-physician collegiality was high, but not when it was low. |
| (Al Barmawi et al., 2019) | Jordan | 228 Critical care and ED RNs | - Pro-QOL-V - Coping Strategies Indicator Questionnaire (CSI) | - Most nurses reported low to average CS, and low to moderate BO and STS levels. They also had low to average problem-solving, social support seeking, and avoidance. Female nurses and those in medical ICUs scored higher on CS. Surgical ICU nurses scored highest on problem-solving and seeking social support, while burn ICU nurses scored highest on avoidance. Positive correlations existed between CSI and CS subscales, while negative correlations were found between avoidance, social support seeking, and STS. Coping strategies were positively associated with CS. Problem-solving explained 27% of CS, and avoidance explained 22.9% of STS. |
| (Alharbi et al., 2020a) | KSA | 516 critical care nurses Saudi and Non-Saudi nurse | - Pro-QOL-V - Pressure injuries, patient falls, and medication errors as the nurse indicators | - Most nurses reported average CS, BO, and STS levels. Low rates of falls and medication errors reflect hands-on care, while pressure injuries indicate non-hands-on care. Hospital characteristics correlated with CS and BO levels, but STS levels were consistent across hospitals. Factors like age, experience, education, staffing, and contractual nature affect nurse performance. Nurse-sensitive indicator rates may relate to CS, BO, or STS levels in Saudi Arabia, suggesting critical care units may foster CF development among nurses |
| (Alharbi et al., 2020b) | KSA | 321 Saudi and non-saudi critical care nurses | - Pro-QOL-V - the Coping strategy indicator (CSI) - The Connor-Davidson Resilience Scale (CD-RISC) | - Critical care nurses had moderate CS, BO, and STS, with 20% reporting very low CS, indicating potential CF. Male gender predicted STS, while being Saudi, holding a master’s degree, and working in ICU or CCU were significant for higher CS. Most scored low on Problem-Solving, and three-quarters used avoidance as a coping strategy. Non-Saudi nurses and females scored higher on Problem-Solving and avoidance. Nurses over 35 and with 16 to 20 years of experience, as well as burn unit nurses, exhibited higher avoidance. Resilience was low, below the US average, with higher resilience in Saudi nurses, those with master's degrees, and CCU/ICU nurses. Positive correlations were found between ProQOL-5 and CD-RISC, and negative correlations between ProQOL-5 and CSI. Resilience explained 66% of CS variance, 26% of BO variance, and 15.4% of STS variance, while coping strategies did not significantly predict any component. |
| (Highfield & Parry-Jones, 2020)Jones | UK | 799 critical care physicians (largest response) | - Pro-QOL-V | - Most participants reported low to average levels of BO and STS, while CS levels were average to high. There were no significant relationships between age, gender, or unit size and ProQOL subscales, indicating these factors are neither predictors nor risk factors for CF. The high CS percentage suggests that the work environment is rewarding |
| (Jo et al., 2020) | Korea | 145 hospice volunteers | - PHQ-9 - Pro-QOL-V | - CS was negatively associated with anxiety and depression, while CF was positively correlated with both. CS mediated the relationship between resilience and anxiety, and CF mediated the relationship between resilience and both anxiety and depression. |
| (Monroe et al., 2020) | USA | 219 critical care nurses | - Pro-QOL-V - The AACN (HWEAT) | - (HWEAT) had a mean composite average of 3.5, indicating an acceptable level. The subscales of "true collaboration," "effective decision making," and "authentic leadership" explained up to 33% of CS. "Authentic leadership" accounted for 22% of BO, while "appropriate staffing," "meaningful recognition," and "authentic leadership" explained 16% of STS. Skilled communication did not explain any ProQOL subscale. |
| (Richardson & Greenle, 2020) | USA | 65 PICU NICU nurses | - Pro-QOL-V | - Participants reported average to high CS and low to average BO and STS. There was no significant relationship between exposure to patient death or near-death experiences and ProQOL among nurses. Education level positively correlated with CS and BO. Experience had a small impact on CS but was significant for BO. Age provided a protective effect, leading to higher CS and lower BO beyond the influence of experience |
| (Salimi et al., 2020) | Iran | 400 ICU nurse | - Pro-QOL-V | - (42%) of subjects had BO scores above average, and (96%) had STS scores above average, indicating unfavorable CF levels. There was a significant positive association between BO and STS, and a significant negative association between CS and BO. Age and years of experience positively correlated with CS levels, while years of experience also positively correlated with both BO and STS levels. NICU nurses had the highest STS levels among critical care nurses. |
| (Weintraub et al., 2020) | USA | 363 pediatric hematology and oncology nurses | - CF and Satisfaction Self-Test for Helpers (CFST) | - A strong positive correlation was found between CF and BO scores (shared variance 53%). Strong negative correlations existed between CS and BO scores (41%) and CF and CS scores (29%). Female sex correlated positively with both CF and BO, but negatively with CS. Factors predicting high CF included female sex, BO score (largest contributor), distress about clinical situations, and teaching on the survey day, explaining 63% of the variance. Participation in administrative activities predicted low CF. CF score, not working on the survey day, and distress about academic stress/administrative burden and coworkers predicted high BO, explaining 71% of the variance. CS score (largest contributor) and socializing with friends/family/pets predicted lower BO. Exercise, socializing, and having nurses for end-of-life care predicted higher CS. CF score, BO score, emotional depletion, and distress about the work environment were significant predictors of lower CS. |
| (Storm & Chen, 2021) | USA | 52 nurses from ICU and Step-down ICU | - Pro-QOL-V - Observation to detect Alarm Fatigue | - 40% of the participants, the majority from ICU were observed to have alarm fatigue - The variable of alarm fatigue was not significantly related to ProQOL subscale |
| (Ageel & Shbeer, 2022) | KSA | 238 CCU nurses 80% RNs | - Pro-QOL-V | - All subscales were within average to high levels. CCU nurses in public hospitals had significantly higher BO levels than those in the private sector. RNs working night shifts experienced significantly higher STS compared to those on day shifts or alternating shifts. |
| (Ahmed et al., 2022) | USA | 375 physicians | - ProQOL-21 - STS scale - BO measure short version - CFA and EFA | - The CFA did not confirm existing models of CF, STS, and BO. EFA revealed four factors—depressed mood, trauma-related issues, and sleep troubles—none representing BO or STS. This suggests that physicians' adverse experiences may not be captured by current definitions of BO, STS, and CF. A qualitative study is needed to explore these findings through physicians' narratives |
| (Aslan et al., 2022) | Turkey | 330 nurses | - Pro-QOL-V - Work‑Related Strain Inventory (WRSI)   Life Attitude Profile (LAP) | - Participants reported low levels of CF, with moderate stress levels. There was a positive correlation between CF and work-related stress, and a negative correlation between work-related strain and life attitude profile. Increased tension negatively affected the meaning of life, and a negative correlation existed between CF and life attitude profile. Females, singles, those with variable shifts, and ICU workers had higher CF levels. Age and years of experience positively correlated with CF. Together, life attitude profile, work-related strain, work manner, years of experience, working unit, and gender explained 35.3% of CF variance. |
| (Calegari et al., 2022). | Italy | 143 physicians and nurses | - Pro-QOL-V - the Coping Orientations to the Problems Experienced | - Nurses had significantly lower BO and STS levels than physicians, with no difference in CS levels. Females more often used "availing social support" and "turning to religion" as coping mechanisms. Professional seniority, a positive attitude, and avoidance coping strategies significantly predicted CS. For BO, predictors included professional seniority, a positive attitude, and problem orientation, while STS was predicted by avoidance, problem orientation, social support, and a positive attitude. |
| (Hameed et al., 2022) | Pakistan | 140 ER and Critical care RN | - Pro-QOL-V | - A positive correlation existed between education level and CF, while a negative correlation was found between years of experience and CS. Income positively correlated with CS. Single nurses reported higher levels of STS compared to married ones. Critical care nurses had average to high levels of CS and low to average levels of CF compared to ER nurses. |
| (Lopez et al., 2022) | USA | 50 ER nurses from 3 hospital during COVID | - Pro-QOL-V | - All nurses had average scores on ProQOL subscales. A positive correlation existed between BO and STS, while a negative correlation was found between BO and CS. There was no significant relationship between STS and CS. Nurses working midshifts had significantly higher BO and STS levels than those on day shifts. Nurses with one child had significantly higher CS subcategory scores than those with two children. |
| (Ma et al., 2022) | China | 342 ED nurses and Physicians | - Pro-QOL-V - Center for Epidemiologic Studies Depression (CES-D) | - Significant differences in depression scores were found based on marital status, professional title, daily work hours, alcohol use, and chronic disease history. Most participants had moderate CS levels, with high levels of depression reported. Only 3% had high CF scores. CS and BO significantly correlated with depressed affect, somatic symptoms, and interpersonal relations. "Being unmarried," chronic disease history, CS, BO, and CF explained 39.6% of the variance in depression. |
| (Nasser et al., 2022) | Egypt | (205) staff nurses who provide care for COVID-19 patients | - Pro-QOL-V - COVID-19 peritraumatic distress index | - Two-thirds of nurses had average levels of CS, BO, and STS, while less than half reported high levels of peritraumatic psychological distress. A significant positive correlation existed between STS and peritraumatic psychological distress. Years of experience strongly predicted CF, along with hospital type and department, which also significantly predicted CF and peritraumatic psychological distress. |
| (Ndlovu et al., 2022). | South Africa | 154 CCU nurse | - Pro-QOL-V | - More than half of the participants reported moderate to high levels of CS, BO, and STS. Positive correlations existed between years of experience and CS, as well as between education level and STS. Nurses caring for only one patient had higher CS and lower BO and STS levels compared to others. |
| (Uzun et al., 2022) | Türkiye | 89 health care workers (nurses, MD, Auxiliary staff) from ICU and non-ICU | - Pro-QOL-V | - A significant positive correlation was found between work areas and working hours with BO. Physicians exhibited higher BO levels than other professionals. Positive correlations exist between education level and both STS and BO. Working in the COVID-19 intensive care unit led to a decrease in physical activity. |
| (Abou Hashish & Ghanem Atalla, 2023) | Egypt | 443 nurses | - Pro-QOL-V - the Coping strategy indicator | - (48.5%) had a moderate level of CS, while the highest percentage reported moderate BO and low STS levels. Nurses moderately used coping strategies such as "social support problem-solving" and "avoidance." A significant positive correlation existed between overall coping strategies and CS, while overall coping strategies were negatively correlated with CF, BO, and STS. As independent variables, coping strategies positively explained 25.6% of the variance in CS and negatively explained 12.4% of CF. CS was negatively correlated with CF and its two dimensions. |
| (Ariapooran & Abdolmaleki, 2023) | Iran | 394 nurses | - Pro-QOL-V - Multi questionnaires | - A multi-questionnaire approach was used in this study, including the "Spiritual Wellbeing (SW) Questionnaire," "Emotional Regulation (ER) Questionnaire," and "Time Perspective Questionnaire." Being female, married, working fixed shifts, and in critical care were predictor factors for high CF. SW, ER, and time perspectives (*Positive Past, Negative Past, Present Hedonistic, Present Fatalistic, and Negative Future)* significantly positively explained CF. |
| (Cicek Korkmaz & Gokoglan, 2023) | Turkey | 451 nurses | - Basic Personality Traits Inventory - The CF-Short Scale | - Personality traits explained 21.9% of CF. A positive correlation existed between neuroticism and CF, while a negative correlation was found between extraversion and CF. Significant negative correlations also existed between CF and the personality traits of extraversion, conscientiousness, agreeableness, and openness to experience |
| (Delle Donne et al., 2023) | Italy | 78 resident physician and specialist physician | - Pro-QOL-V - DASS-21 | - MDs experienced mild to moderate depression (25.6%), anxiety, and stress (5.1%), with medium levels of CF (BO 94.9%, STS 61.5%) and CS (74.4%). MD specialty positively correlated with CS and negatively with BO. Residents had mild to moderate anxiety and stress compared to specialists, with those having children showing higher CS and anxiety. MDs with medium BO and mild to moderate anxiety perceived lower team support. Female specialists had higher BO, STS, depression, anxiety, and stress, and lower CS than males |
| (Jeong & Shin, 2023) | South Korea | 147 ICU nurse | - Pro-QOL-V   (only STS scale)   - Maslach BO Inventory - The resilience scale | - 83% of participants reported average STS, while (75%) indicated high-level BO. BO significantly differed based on gender, age, marital status, years of experience, and monthly wages. Females, nurses in their 20s, unmarried nurses, those with less than five years of experience, and low-wage nurses reported significantly higher BO. Resilience also differed significantly based on marital status, religion, and monthly wage; married nurses, those who were religious, and high-wage earners reported significantly higher resilience. Resilience was a significant mediator between critical care nurses' STS and BO. |
| (Juniarta et al., 2023) | Indonesia | 234 RNs working with covid | - Pro-QOL-V - Self-compassion scale | - Participants reported high-level CS, moderate STS, and moderate BO. High means were observed in the SCS for “common humanity,” “mindfulness,” and “self-kindness.” A positive relationship existed between CS and lower correlations of BO with higher levels of “self-kindness,” “common humanity,” and “mindfulness,” as well as lower levels of “self-judgment” and “isolation.” STS was strongly positively correlated with “self-judgment” and “isolation.” There was a negative correlation between both STS and BO with lower levels of “self-kindness,” “humanism,” and “mindfulness self-compassion.” |
| (Panagou et al., 2023) | Greece | 147 PICU MD &Nurse | - Pro-QOL-V - Professional-life characteristics questionnaire | - Nurses reported moderate levels of STS and BO, with high to average levels of CS. One-third of respondents indicated that “patient death” significantly affects them during their shift. Married PICU health professionals exhibited higher levels of STS and overprotective attitudes toward their families. PICU MDs reported higher levels of CS and lower BO than nurses, while trainee MDs showed higher levels of BO. Nurses expressed “low job security” and dissatisfaction with wages and working conditions, which may contribute to a decrease in CS. A positive correlation existed between education level and CS, while a negative correlation was found with BO |
| (Ruba Sam et al., 2023) | UAE | 525 Nurses ER, OR, ICU, Medical and surgical units | - Pro-QOL-V | - The prevalence of moderate levels of CF in nurses is 67.6% for BO and 54.7% for STS. Factors such as age, gender, ethnicity, level of education, marital status, job stress experience, hospital type, and work specialty significantly predicted CS. Similarly, ethnicity, age, marital status, level of education, years of experience, workplace, specialty, and job stress experience significantly predicted BO. Ethnicity, designation, workplace, and job stress experience significantly predicted STS. A significant negative correlation exists between CS and BO, as well as between CS and STS, while a positive correlation exists between STS and BO. |
| (Subih et al., 2023) | Jordan | 203 ER nurses | - Pro-QOL-V | - A + negligeable relationship between CS and “Level of Education” - A - negligible correlation between STS and comorbidity |
| (Unlugedik & Akbas, 2023) | Turkey | 167 Critical care nurses | - The Spiritual Well-Being Scale - The CF-Short Scale | - A negative correlation exists between level of education and spiritual well-being. A high mean CF score was observed, with youth, few years of experience, and being single identified as predictors for high CF. No differences in CF levels were found based on educational level. Well-being has a positive and statistically significant impact on CF, BO, and STS. |
| (Zeng et al., 2023) | China | 520 new nurses from different wards | - Pro-QOL-V | - Factors predicting higher CS scores include being a new nurse, working day shifts, job satisfaction, sleeping over 7 hours, being a non-smoker, and non-alcoholic. Higher BO scores are predicted by being a new nurse, single status, working night shifts, over 8 hours per day, low job satisfaction, sleeping under 7 hours, and experiencing workplace violence. STS is predicted by being a new nurse, working over 8 hours, and experiencing workplace violence. - Job satisfaction, sleeping over 7 hours, smoking, and workplace violence explained 22.1% of CS; job satisfaction, sleeping over 7 hours, and daily work hours explained 38.3% of BO. Workplace violence and work hours explained 2.1% of STS. Overall, nurses reported low to moderate CS prevalence (93.65%) and moderate to high BO (85.38%) and STS (83.46%) |
