# Supplementary material for: Compassion Fatigue Among Critical Care Nurses and Physicians: A Scoping Review
Source: Nurs Open. 2025 Dec 23;12(12):e70410. doi: 10.1002/nop2.70410 (PMC12723723; doi:10.1002/nop2.70410)
Supplement: Supplementary file 2 — Table S2: Summary table of included mixed method studies. [file NOP2-12-e70410-s004.docx]

**Table 2. Summary table of included Mixed Method studies**

| **Author** | **Country** | **Sample** | **Measurement tool** | **Overall findings** |
| --- | --- | --- | --- | --- |
| (Milligan & Almomani, 2020) | Qatar | 81 ICU nurses  8 nurses for QUAL | - The Death Attitude Profile Revised (DAP-R) - Thematic qualitative analysis | - "Fear of death" and "death avoidance" were positively correlated with qualifications, gender, and years in the ICU, but negatively correlated with time employed in the organization. "Fear of death" negatively correlated with age, while "death avoidance" positively correlated with age. Female gender and longer work experience in critical care were identified as risk factors for CF. - Qualitative outcomes: **Theme 1:** Compassionate care was often not delivered in critical care units, as the focus was primarily on competent care; **Theme 2:** There was a desire to provide competent care, but barriers such as staff shortages and high patient turnover were identified. |
| (Pehlivan & Guner, 2020) | Turkey | 16 nurse managers | - Pro-QOL-IV - Perceived Stress Scale - Resilience scale - CFRP questionnaire (qualitative data) | - Baseline mean scores indicated that nurse managers had moderate CF and CS, with low BO. The CFRP significantly reduced CF and stress in nurse managers but did not lead to significant changes in CS, BO, or resilience. While the CFRP did not significantly reduce BO, it increased job satisfaction and professional awareness among nurse managers. Nurses reported that the CFRP was useful for coping with stress. Improving coping and communication skills within the nurse management team can positively impact the quality of patient care |
| (Christianson et al., 2023) | USA | 1224 RN and 68 LPN | - Copenhagen BO Inventory WRB scale - CF Short Scale (BO and VT) - Intention to Leave the Profession (Qualitative question) | - Nurses were categorized into three groups: intend to leave (ITL), intend to stay (ITS), or uncertain. The ITL group exhibited higher BO compared to the ITS group. There was a significant difference in overall CFSS scores and both subscales (BO and VT) between the ITL groups. ITL among nurses practicing during the COVID-19 pandemic correlated with higher WRB and CF scores. Although WRB and CF were significantly higher in COVID nurses than in non-COVID nurses, COVID nurses were less likely to indicate ITL, possibly due to income disparities. WRB was a significant variable for both the uncertain and ITL groups, with odds ratios (OR) of 1.034 and 1.047, respectively. CF, COVID patient management, and practice type were significant factors in the ITL group |
