# Supplementary material for: Compassion Fatigue Among Critical Care Nurses and Physicians: A Scoping Review
Source: Nurs Open. 2025 Dec 23;12(12):e70410. doi: 10.1002/nop2.70410 (PMC12723723; doi:10.1002/nop2.70410)
Supplement: Supplementary file 3 — Table S3:Summary table of included qualitative studies. [file NOP2-12-e70410-s003.docx]

**Table 3. Summary table of included Qualitative studies**

| **Author** | **Country** | **Sample** | **Design** | **Overall findings** |
| --- | --- | --- | --- | --- |
| (Jakimowicz et al., 2018b) | Australia | 21 critical care nurses | Qualitative Charmaz Grounded theory | - Study examined the relationships between CCS, CF, and patient-centered nursing in intensive care, revealing their interconnectedness. Nurses balance their passion for critical care with stress and difficulties, navigating a compassion continuum between satisfaction and fatigue. A core category of "Expectations" emerged, including:   **Regulatory Body**: Governance of practice.  **Place of Work**: Management expectations for patient-centered care and collaboration.  **Colleagues**: Expectations for competence and support.  **Self: Importance** of meeting various expectations.  Key themes included:  **Life in the Balance**: Tension between biomedical skills and compassionate care.  **Keep My Patient Alive**: Central to the nursing role.  **Stretching Versus Overstretching**: Balancing expertise without overextending.  **Passion and Pressure**: Rewarding work amid constant pressure.  **Understanding and Advocacy:** Satisfaction and fatigue linked to care delivery.  **Tenacity and Fragility:** Managing emotions to maintain professionalism amidst challenges |
| (Andrews et al., 2020) | UK | 29 critical care nurses | constructivist grounded theory | - The core concept emerging from the data analysis was "Needing Permission," specifically for self-compassion, which nurses sought from themselves and others. This was informed by three concepts:   **Hardwired to be Caregivers:** Reflects the innate motivation to nurse and how it aligns with their identity.  **Needing a Stable Base:** Nurses require a sense of safety and security in their workplace to practice self-care and compassion, influenced by factors like job security and effective leadership.  **Managing the Emotions of Caring:** Highlights the emotional complexities involved in caregiving.   - Participants expressed a strong desire to care for others to feel valued. The framework situates permission as central to enabling nurses to care for themselves, linking it to emotional management. Findings indicated that nurses face uncertainty due to NHS changes, such as service restructuring and staffing issues, which destabilize their ability to be self-caring and compassionate. - Participants advocated for a holistic approach to self-care, recognizing the need to address their well-being but feeling unable to act on early warning signs of illness due to the lack of internal or external permission. |
| (Gustafsson & Hemberg, 2022) | Finland | 7 nurses | Qualitative explorative design | - The analysis generated five themes:   **Compassion as an Empathic Gift and CF as Compassion Overload:** Highlights the dual nature of compassion and its potential to lead to burnout.  **CF as Exhausting the Nurse in Professional and Private Life:** Emphasizes the toll of compassion fatigue on both personal and professional well-being.  **CF as a Crisis with Potentially Valuable Insights:** Suggests that experiences of compassion fatigue can provide important lessons.  **CF Can Be Managed Through Self-Care and Focus on Self:** Advocates for self-care strategies as a means to cope with compassion fatigue.  **CF is Affected by Life Itself and Multifaceted Factors**: Acknowledges that various life circumstances and factors influence compassion fatigue |
