# Supplementary material for: Compassion Fatigue Among Critical Care Nurses and Physicians: A Scoping Review
Source: Nurs Open. 2025 Dec 23;12(12):e70410. doi: 10.1002/nop2.70410 (PMC12723723; doi:10.1002/nop2.70410)
Supplement: Supplementary file 4 — Table S4: Summary table of included experimental studies. [file NOP2-12-e70410-s002.docx]

**Table 4. Summary table of included Experimental studies**

| **Author** | **Country** | **Sample** | **Measurement tool** | **Design** | **Overall findings** |
| --- | --- | --- | --- | --- | --- |
| (Kharatzadeh et al., 2020) | Iran | 60 critical care nurses | - Pro-QOL-V - Cognitive Emotion Regulation Questionnaire (CERQ) - Depression, Anxiety, Stress Scale (DASS-21) | RCT | - The study on the effect of the emotional training session on PROQOL found that, at baseline, there were no differences between groups in CERQ, DASS-21, and ProQoL-5 subscale scores. After the intervention, the treatment group experienced a significant increase in compassion satisfaction (CS) and a significant decrease in burnout (BO), although there was no significant change in compassion fatigue (CF) levels between the two groups. The high CS subcategory in the treatment group rose from 53.8% to 57.7%, while the average BO decreased from 46.2% to 30.8%, and high CF dropped from 3.8% to 0%. Significant improvements were noted in four of the five adaptive aspects of cognitive coping strategies, alongside substantial reductions in depression, anxiety, and stress within the intervention group. However, there were no significant differences in the four maladaptive aspects of the CERQ when comparing the treatment group with wait-list controls. |
| (Arbios et al., 2022) | USA | Between 149 and 168 PICU nurses | Self-Reporting Questionnaire-20 | Quality improvement study | - The study on Cumulative Stress Debriefing (CSD) found that attendees were less likely to consider leaving their jobs or the nursing profession compared to non-attendees. A higher percentage of CSD participants reported moderate to high job satisfaction at 9 and 12 months and enjoyed their work more at all follow-up points. Attendees also experienced less unhappiness, tiredness, and crying over time. Initially, rates of nervousness, stress, and poor-quality sleep were higher among attendees but decreased to lower levels than non-attenders by 9 and 12 months. Overall, the results indicated a correlation between CSD attendance and reduced compassion fatigue symptoms. |
| (Pehlivan Saribudak et al., 2023) | Turkey | 125 oncology hematology nurses | - Pro-QOL-V - Perceived stress scale - Resilience scale | RCT | The study on the effects of short- and long-term Compassion Fatigue Resilience Program (CFRP) found no significant differences in mean CF scores among short-term, long-term, or control groups. However, CF scores at the 3, 6, and 12-month follow-ups were significantly higher than pre-test scores. Long-term training showed significant pre- and post-test differences for the control group.   - For BO, no group differences were found, but follow-up scores were significantly higher than pre-test scores. CS showed significant differences, with initiative groups reporting higher scores, and follow-up scores were also significantly higher than pre-tests. No significant differences were observed for psychological safety (PS) and resilience regarding group effects, although resilience showed significant time-related changes. |
| (Asadollah et al., 2023) | Iran | 66 NICU RN in 5 NICUs | - Nursing CF Inventory (NCFI). | RCT | **The Impact of Loving-Kindness Meditation LKM on CF**   - The study on the impact of LKM on CF involved an intervention group that received meditation audios via WhatsApp and interacted with the researcher, encouraged to listen at least three times a week. The control group also had a WhatsApp group but received updates on improving mental health during COVID. - Before the intervention, CF levels were similar in both groups. However, post-intervention, the mean CF in the intervention group significantly decreased, with p-values of 0.02 for within-group differences and 0.001 for between-group differences. Overall, LKM led to a reduction in CF scores among nurses. |
| (Egami & Highfield, 2023) | USA | 22 regular, &traveler RNs working in NICU hospital | - Pro-QOL-V - Mindfulness Attention Awareness Scale (MAAS) | Single group Pre-Post test | - The study tested whether self-guided mindfulness using the Premium Moodfit app improved NICU nurses' professional QOL. Nine RNs submitted pre- and post-tests and used Moodfit; two practiced yoga or meditation, but their MAAS scores were unchanged. - The hypothesis was partially supported, showing a decrease in STS, but no changes in BO or CS |
